# Association Between Preserved Ratio Impaired Spirometry and COPD With Coronary Artery Calcification Measured by Coronary CT Scan

**DOI:** 10.1016/j.chest.2025.02.030

**Published:** 2025-03-08

**Authors:** Purnadeo N. Persaud, Lauren S. Munoz Tremblay, Yanjun Wu, Peter Oro, Daniel A. Culver, James K. Stoller, Xiaofeng Wang, Raul Seballos, Richard H. Cartabuke, Trishul Siddharthan, Vickram Tejwani

**Affiliations:** a Department of Pulmonary and Critical Care Medicine, Integrated Hospital Care Institute, Cleveland Clinic, Cleveland, OH; b Division of Pulmonary and Critical Care Medicine, University of Washington, Seattle, WA; c Quantitative Health Sciences, Cleveland Clinic Lerner College of Medicine, Cleveland, OH; d Department of Internal Medicine, South Pointe Hospital—Cleveland Clinicl, Cleveland, OH; e Education Institute, Cleveland Clinic, Cleveland, OH; f Department of Executive Health, Cleveland Clinic, Cleveland, OH; g Division of Pulmonary and Critical Care Medicine, University of Miami, Miami, FL

To the Editor:

The association between COPD and coronary artery disease (CAD) is well established, with COPD as an independent risk factor.^1^ Studies suggest that preserved ratio impaired spirometry (PRISm), defined by FEV_1_ < 80% predicted and FEV_1_/FVC ≥ 70%, may precede COPD.^2^ PRISm has been linked to elevated cardiovascular-related mortality; however, the cause remains unclear.^3^

Coronary artery calcification score (CACS), using coronary CT, is a growing CAD indicator.^4^ Studies have associated COPD with higher CACS; however, data for PRISm are limited.^1^^,^^5^ We hypothesize that PRISm is associated with elevated CACS, consistent with the notion that CAD helps explain increased cardiovascular mortality in patients with COPD. This study investigates the association between PRISm, COPD, and CACS, to better understand the complex relationship between pulmonary and cardiovascular health.

## Methods

### Study Population

This retrospective cohort study used data from individuals enrolled in our Executive Health program (2000-2023), which includes comprehensive screening with spirometry and coronary CT scan, independent of risk factors. CACS measurements were conducted as outlined by Agatston et al.^6^ Inclusion criteria included pulmonary function testing (PFT), coronary CT scan, and an outpatient screening visit, all within 14 days. Patients with cardiac symptoms prompting testing or unknown smoking histories were excluded. This study was approved by the Cleveland Clinic’s institutional review board [#23-161].

### Data Collection

Patients were categorized based on spirometry: normal (FEV_1_/FVC ≥ 0.7 and FEV_1_ ≥ 80% predicted), PRISm (FEV_1_/FVC ≥ 0.7 and FEV_1_ < 80% predicted), and COPD (FEV_1_/FVC < 0.7). Reference PFT prediction values were from the Hankinson 1999 National Health and Nutrition Examination Survey (NHANES) and the Global Lung Initiative (GLI) race-neutral equations.^7^ We employed both equations to address variability in race-stratified models given these equations affect disease classification.^8^ Demographic data, BMI, comorbidities, and smoking history were obtained.

### Statistical Analyses

An ordinal logistic regression model adjusted for age (< 45 or ≥ 45 years old), sex, smoking status/pack-years, BMI, diabetes, hypertension, and dyslipidemia was used to study the association between CACS and PRISm/COPD, where proportional odds assumption was met. CACS was categorized into 0, 1-99, and ≥ 100. Logistic regression estimated the probability of non-zero CACS, with ORs and 95% CI. Significance was defined as *P* < .05 (2-tailed). Analyses were conducted using R software (version 4.2.0; R Foundation for Statistical Computing).

## Results

Three thousand five hundred seventy-three participants were included (5,229 participants were screened: 1,611 were excluded because not all tests were performed within 14 days, 5 for cardiac symptoms, and 40 for unknown smoking status). Using NHANES, 292 patients had COPD (8.2%), 258 had PRISm (7.2%), 3,023 had normal PFTs (84.6%). Using GLI race-neutral values, 292 had COPD, 140 had PRISm, and 3,141 had normal PFTs (Table 1).

**Table 1:** Patient Demographics Based on NHANES And GLI Race-Neutral Reference Equations

NHANES	Normal (n = 3,023)	PRISm (n = 258)	COPD (n = 292)	*P*^a^
Age				**.008**
< 45	432 (14.3)	29 (11.2)	24 (8.2)	
≥ 45	2,591 (85.7)	229 (88.8)	268 (91.8)	
Sex				.521
Female	887 (29.3)	72 (27.9)	77 (26.4)	
Male	2,136 (70.7)	186 (72.1)	215 (73.6)	
Race				**< .001**
White	2,564 (84.8)	179 (69.4)	244 (83.6)	
Black	69 (2.3)	3 (1.2)	6 (2.1)	
Asian	62 (2.1)	31 (12)	8 (2.7)	
Other^b^	328 (10.9)	45 (17.4)	34 (11.6)	
BMI				**< .001**
Normal	858 (28.4)	49 (19)	103 (35.3)	
Obese	828 (27.4)	102 (39.5)	60 (20.5)	
Overweight	1,319 (43.6%)	106 (41.1%)	129 (44.2%)	
Underweight	18 (0.6)	1 (0.4)	0 (0)	
Smoking status				**.046**
Never	2,295 (75.9)	192 (74.4)	200 (68.5)	
Former	568 (18.8)	47 (18.2)	70 (24)	
Current	160 (5.3)	19 (7.4)	22 (7.5)	
Comorbidity count				
Mean ± SD	0.8 ± 1.0	1.3 ± 1.3	0.9 ± 1.1	**< .001**
Median (IQR)	0.0 (0.0-1.0)	1.0 (0.0-2.0)	1.0 (0.0-2.0)	**< .001**
CHF	18 (0.6)	7 (2.7)	5 (1.7)	**< .001**
Pulmonary circulation disorders	8 (0.3)	2 (0.8)	1 (0.3)	.362
Liver disease	152 (5)	26 (10.1)	12 (4.1)	**.002**
Sleep apnea	484 (16)	66 (25.6)	47 (16.1)	**< .001**
Diabetes	127 (4.2)	34 (13.2)	12 (4.1)	**< .001**
GERD	460 (15.2)	56 (21.7)	53 (18.2)	**.013**
PUD	53 (1.8)	4 (1.6)	3 (1)	.645
HTN	667 (22.1)	87 (33.7)	67 (22.9)	**< .001**
PVD	180 (6)	22 (8.5)	23 (7.9)	.135
Asthma	148 (4.9)	25 (9.7)	51 (17.5)	**< .001**
Dyslipidemia	1,624 (53.7)	169 (65.5)	162 (55.5)	**.001**
COPD GOLD stage				
1	NA	NA	182 (62.3)	NA
2	NA	NA	101 (34.6)	NA
3	NA	NA	9 (3.1)	NA
CAC score, mean ± SD	78.3 ± 306.2	93.8 ± 256.5	120.9 ± 350.4	.065
CAC score, Median (IQR)	0.0 (0.0-24.0)	0.0 (0.0-38.0)	0.3 (0.0-58.0)	**< .001**
CAC score				**< .001**
0	1,836 (60.7)	144 (55.8)	143 (49)	
1-99	731 (24.2)	62 (24)	85 (29.1)	
≥100	456 (15.1)	52 (20.2)	64 (21.9)	

GLI Race-Neutral	Normal (n = 3,141)	PRISm (n = 140)	COPD (n = 292)	*P*
Age				
< 45	450 (14.3)	11 (7.9)	24 (8.2)	**.002**
≥ 45	2,691 (85.7)	129 (92.1)	268 (91.8)	
Sex				
Female	921 (29.3)	38 (27.1)	77 (26.4)	.503
Male	2,220 (70.7)	102 (72.9)	215 (73.6)	
Race				**< .001**
White	2,658 (84.6)	85 (60.7)	244 (83.6)	
Black	60 (1.9)	12 (8.6)	6 (2.1)	
Asian	75 (2.4)	18 (12.9)	8 (2.7)	
Other^b^	348 (11.1)	25 (17.9)	34 (11.6)	
BMI				**< .001**
Normal	884 (28.1)	23 (16.4)	103 (35.3)	
Obese	867 (27.6)	63 (45)	60 (20.5)	
Overweight	1,371 (43.6)	54 (38.6)	129 (44.2)	
Underweight	19 (0.6)	0 (0)	0 (0)	
Smoking status				
Never	2,381 (75.8)	106 (75.7)	200 (68.5)	**.026**
Former	593 (18.9)	22 (15.7)	70 (24)	
Current	167 (5.3)	12 (8.6)	22 (7.5)	
Comorbidity count				
Mean ± SD	0.8 ± 1.0	1.4 ± 1.3	0.9 ± 1.1	**< .001**
Median (IQR)	0.0 (0.0-1.0)	1.0 (0.0-2.0)	1.0 (0.0-2.0)	**< .001**
CHF	20 (0.6)	5 (3.6)	5 (1.7)	**< .001**
Pulmonary circulation disorders	8 (0.3)	2 (1.4)	1 (0.3)	**.049**
Liver disease	166 (5.3)	12 (8.6)	12 (4.1)	.150
Sleep apnea	509 (16.2)	41 (29.3)	47 (16.1)	**< .001**
Diabetes	141 (4.5)	20 (14.3)	12 (4.1)	**< .001**
GERD	487 (15.5)	29 (20.7)	53 (18.2)	.143
PUD	55 (1.8)	2 (1.4)	3 (1)	.637
HTN	703 (22.4)	51 (36.4)	67 (22.9)	**< .001**
PVD	187 (6)	15 (10.7)	23 (7.9)	**.039**
Asthma	156 (5)	17 (12.1)	51 (17.5)	**< .001**
Dyslipidemia	1,703 (54.2)	90 (64.3)	162 (55.5)	.062
COPD GOLD stage				
1	NA	NA	209 (71.6)	NA
2	NA	NA	76 (26)	NA
3	NA	NA	7 (2.4)	NA
CAC score, mean ± SD	79.8 ± 306.7	74.3 ± 188.4	120.9 ± 350.4	.086
CAC score, median (IQR)	0.0 (0.0-24.0)	0.0 (0.0-37.5)	0.3 (0.0-58.0)	**< .001**
CAC score				**< .001**
0	1,899 (60.5)	81 (57.9)	143 (49)	

GLI Race-Neutral	Normal (n = 3,141)	PRISm (n = 140)	COPD (n = 292)	*P*
1-99	763 (24.3)	30 (21.4)	85 (29.1)	
≥ 100	479 (15.2)	29 (20.7)	64 (21.9)	

All data are presented as No. (%) unless indicated otherwise. Boldface indicates *P* < .05. CAC = coronary artery calcification; CHF = congestive heart failure; GERD = gastroesophageal reflux disease; GLI = Global Lung Initiative; GOLD = Global Initiative for Chronic Obstructive Lung Disease; HTN = hypertension; NA = not applicable; NHANES = National Health and Nutrition Examination Survey; IQR = interquartile range; PRISm = preserved ratio impaired spirometry; PUD = peptic ulcer disease; PVD = peripheral vascular disease.

a *P* values represent comparisons between all 3 groups.

b “Other” includes American Indian/Alaska Native, Native Hawaiian/Pacific Islander, Multiracial/Multicultural, Declined, Unavailable, and Unknown.

Patients with COPD were older than those with normal spirometry, but not those with PRISm (NHANES: *P* = .008, GLI race-neutral: *P* = .002). Higher BMI was observed in patients with PRISm compared with those with COPD or normal spirometry (NHANES and GLI race-neutral: *P* < .001). Smoking status differed between groups (Table 1).

In unadjusted models using NHANES, median (interquartile range) CACS was highest in COPD (0.3 [0-58.0]), followed by PRISm (0 [0-38.0]) and normal spirometry (0 [0-24.0]) (*P* < .001). Using GLI race-neutral values, median CACS was highest in COPD (0.3 [0-58.0]), followed by PRISm (0 [0-37.5]) and normal spirometry (0 [0-24.0]) (*P* < .001).

**Figure 1:**
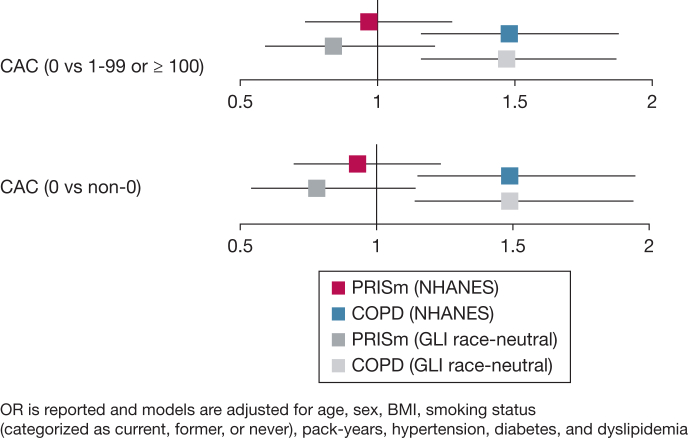
Association of PRISm and COPD with CACS. CAC = coronary artery calcification; CACS = coronary artery calcification score; GLI = Global Lung Initiative; NHANES = National Health and Nutrition Examination Survey; PRISm = preserved ratio impaired spirometry.

In adjusted models using NHANES, individuals with COPD had significantly higher odds of CACS 1-99 or ≥ 100 (OR, 1.48; 95% CI, 1.16-1.88; *P* = .001) (Fig 1). This relationship was not observed in PRISm (OR, 0.97; 95% CI, 0.74-1.27; *P* = .817). Using GLI race-neutral equations, similar findings were identified for COPD (OR, 1.47; 95% CI, 1.16-1.87; *P* = .002), with no association in PRISm (OR, 0.84; 95% CI, 0.59-1.21; *P* = .355).

Additionally, using NHANES, COPD was associated with higher odds of CACS > 0 (OR, 1.49; 95% CI, 1.15-1.95; *P* = .003), whereas PRISm showed no association (OR, 0.93; 95% CI, 0.70-1.23; *P* = .612). Similar associations were seen using GLI-race neutral equations for COPD (OR, 1.49; 95% CI, 1.14-1.94; *P* = .003) and PRISm (OR, 0.78; 95% CI, 0.54-1.14; *P* = .207) (Fig 1).

## Discussion

We found an association between COPD and elevated CACS, but not in PRISm. The results also highlight that race-neutral spirometry equations affect disease classification interpretation.

Our COPD and CACS association is consistent with that of prior studies,^1^ despite our healthy cohort screening population. A recent study showed that PRISm was associated with elevated CACS, but this was only seen in people who had smoked.^9^ Another study showed that CACS progression rates over 5 years were higher in PRISm compared with those with normal spirometry.^5^ Our findings may have differed because of the study’s cross-sectional design and different study population, which included a low number of people who had ever smoked and higher BMIs.

Several limitations of this study warrant comment. First, the study population was recruited from an executive health program composed of predominantly White male individuals with minimal comorbidities, limiting generalizability. Second, the analysis was solely cross-sectional. Our study lacked post-bronchodilator spirometry, which could impact the COPD and PRISm classification. Our study included patients from 2000 to 2023, with the effect of advancements in CT on CACS not taken into consideration. Furthermore, differences in prediction equations could contribute to the reduced sample size of participants with PRISm, rather than a biological reclassification of participants. A strength of this study is that asymptomatic individuals were included, thereby obviating the confounding and biases introduced when symptomatic patients are recruited.

The lack of association between PRISm and CACS may suggest that alternative causes could explain the increased cardiovascular events previously reported. For example, noncalcified atherosclerosis may not be apparent with coronary CT scan.^4^ Possibly, COPD and PRISm could lead to cardiovascular events through separate mechanisms, suggesting that PRISm may be a heterogenous entity with subtypes—potentially based on BMI status and smoking history, which we were underpowered to explore.^9^^,^^10^ Obesity, with well-documented effects on small airway function, could also confound the relationship between spirometry values and CACS.

Our study highlights the need for larger studies and further research on the risk factors influencing CAD in PRISm. Future studies should explore the similarities and differences between COPD and PRISm and other potential causes of cardiovascular risk in PRISm.

## Funding/Support

This work was directly funded by a 10.13039/100007311Cleveland Clinic Executive Health grant. V. T. is supported by 10.13039/100000050National Institutes of Health National Heart, Lung, and Blood Institute K23HL173570.

## Financial/Nonfinancial Disclosures

None declared.
